# VAV3 mediates resistance to breast cancer endocrine therapy

**DOI:** 10.1186/bcr3664

**Published:** 2014-05-28

**Authors:** Helena Aguilar, Ander Urruticoechea, Pasi Halonen, Kazuma Kiyotani, Taisei Mushiroda, Xavier Barril, Jordi Serra-Musach, Abul Islam, Livia Caizzi, Luciano Di Croce, Ekaterina Nevedomskaya, Wilbert Zwart, Josefine Bostner, Elin Karlsson, Gizeh Pérez Tenorio, Tommy Fornander, Dennis C Sgroi, Rafael Garcia-Mata, Maurice PHM Jansen, Nadia García, Núria Bonifaci, Fina Climent, María Teresa Soler, Alejo Rodríguez-Vida, Miguel Gil, Joan Brunet, Griselda Martrat, Laia Gómez-Baldó, Ana I Extremera, Agnes Figueras, Josep Balart, Robert Clarke, Kerry L Burnstein, Kathryn E Carlson, John A Katzenellenbogen, Miguel Vizoso, Manel Esteller, Alberto Villanueva, Ana B Rodríguez-Peña, Xosé R Bustelo, Yusuke Nakamura, Hitoshi Zembutsu, Olle Stål, Roderick L Beijersbergen, Miguel Angel Pujana

**Affiliations:** 1 Breast Cancer and Systems Biology Unit, Translational Research Laboratory, Catalan Institute of Oncology (ICO), Bellvitge Institute for Biomedical Research (IDIBELL), Avda. Gran via 199, L’Hospitalet del Llobregat, Barcelona 08908, Catalonia, Spain; 2 Division of Molecular Carcinogenesis, Center for Biomedical Genetics and Cancer Genomics Centre, The Netherlands Cancer Institute, Plesmanlaan 121, Amsterdam 1066 CX, The Netherlands; 3 Center for Genomic Medicine, RIKEN, 1-7-22 Suehiro-cho, Tsurumi-ku, Yokohama City, Kanagawa 230-0045, Japan; 4 Department of Physical Chemistry, Institute of Biomedicine (IBUB), Avda. Diagonal 643, University of Barcelona, Barcelona 08028, Catalonia, Spain; 5 Catalan Institution for Research and Advanced Studies (ICREA), C/ Lluís Companys 23, Barcelona 08010, Catalonia, Spain; 6 ICO, Girona Biomedical Research Institute (IDIBGI), Hospital Josep Trueta, Avda. França s/n, Girona 17007, Catalonia, Spain; 7 Department of Genetic Engineering and Biotechnology, University of Dhaka, Dhaka 1000, Bangladesh; 8 Centre for Genomic Regulation (CRG), C/ Dr. Aiguader 88, Barcelona 08003, Catalonia, Spain; 9 Universitat Pompeu Fabra (UPF), C/ Dr. Aiguader 88, Barcelona 08003, Catalonia, Spain; 10 Department of Molecular Pathology, The Netherlands Cancer Institute, Plesmanlaan 121, Amsterdam 1066 CX, The Netherlands; 11 Department of Clinical and Experimental Medicine, Division of Oncology, Linköping University, County Council of Östergötland, Sandbäcksgatan 7, Linköping SE-58185, Sweden; 12 Department of Oncology, Karolinska University Hospital, Stockholm South General Hospital, Sjukhusbacken 10, Stockholm SE-11883, Sweden; 13 Department of Pathology, Molecular Pathology Research Unit, Massachusetts General Hospital, 13th St. Charlestown, Boston, MA 02129, USA; 14 Department of Cell Biology and Physiology, University of North Carolina at Chapel Hill, 111 Mason Farm Rd., Chapel Hill, NC 27599-7545, USA; 15 Department of Medical Oncology, Erasmus University Medical Center, Cancer Institute, PO Box 2040, Rotterdam 3000 CA, The Netherlands; 16 Translational Research Laboratory, ICO, IDIBELL, Avda. Gran via 199, L’Hospitalet del Llobregat, Barcelona 08908, Catalonia, Spain; 17 Department of Pathology, University Hospital of Bellvitge, IDIBELL, Avda. Feixa Llarga s/n, L’Hospitalet del Llobregat, Barcelona 08908, Catalonia, Spain; 18 Department of Medical Oncology, Breast Cancer Unit, ICO, IDIBELL, Avda. Gran via 199, L’Hospitalet del Llobregat, Barcelona 08908, Catalonia, Spain; 19 Lombardi Comprehensive Cancer Center, Georgetown University Medical Center, 3970 Reservoir Rd., Washington, DC 20057, USA; 20 Department of Molecular and Cellular Pharmacology, University of Miami, Miller School of Medicine, 1600 NW 10th Ave., Miami, FL 33136, USA; 21 Department of Chemistry, University of Illinois, 505 South Mathews Ave., Urbana, IL 61801, USA; 22 Cancer Epigenetics and Biology Program (PEBC), IDIBELL, Avda. Gran via 199, L’Hospitalet del Llobregat, Barcelona 08908, Catalonia, Spain; 23 Department of Physiological Sciences II, School of Medicine, University of Barcelona, Avda. Feixa Llarga s/n, L’Hospitalet del Llobregat, Barcelona 08908, Catalonia, Spain; 24 Cancer Research Center (CSIC), University of Salamanca, Campus Miguel de Unamuno, Salamanca 37007, Spain; 25 Laboratory of Molecular Medicine, Human Genome Center, Institute of Medical Science, The University of Tokyo, 4-6-1 Shirokanedai, Minato-ku, Tokyo 108-8639, Japan; 26 Present address: Onkologikoa Foundation, Biodonostia, San Sebastián, Doctor Begiristain 121, Guipúzcoa 20014, Spain

## Abstract

**Introduction:**

Endocrine therapies targeting cell proliferation and survival mediated by estrogen receptor α (ERα) are among the most effective systemic treatments for ERα-positive breast cancer. However, most tumors initially responsive to these therapies acquire resistance through mechanisms that involve ERα transcriptional regulatory plasticity. Herein we identify VAV3 as a critical component in this process.

**Methods:**

A cell-based chemical compound screen was carried out to identify therapeutic strategies against resistance to endocrine therapy. Binding to ERα was evaluated by molecular docking analyses, an agonist fluoligand assay and short hairpin (sh)RNA–mediated protein depletion. Microarray analyses were performed to identify altered gene expression. Western blot analysis of signaling and proliferation markers, and shRNA-mediated protein depletion in viability and clonogenic assays, were performed to delineate the role of VAV3. Genetic variation in *VAV3* was assessed for association with the response to tamoxifen. Immunohistochemical analyses of VAV3 were carried out to determine its association with therapeutic response and different tumor markers. An analysis of gene expression association with drug sensitivity was carried out to identify a potential therapeutic approach based on differential VAV3 expression.

**Results:**

The compound YC-1 was found to comparatively reduce the viability of cell models of acquired resistance. This effect was probably not due to activation of its canonical target (soluble guanylyl cyclase), but instead was likely a result of binding to ERα. VAV3 was selectively reduced upon exposure to YC-1 or ERα depletion, and, accordingly, VAV3 depletion comparatively reduced the viability of cell models of acquired resistance. In the clinical scenario, germline variation in *VAV3* was associated with the response to tamoxifen in Japanese breast cancer patients (rs10494071 combined *P* value = 8.4 × 10^−4^). The allele association combined with gene expression analyses indicated that low VAV3 expression predicts better clinical outcome. Conversely, high nuclear VAV3 expression in tumor cells was associated with poorer endocrine therapy response. Based on *VAV3* expression levels and the response to erlotinib in cancer cell lines, targeting EGFR signaling may be a promising therapeutic strategy.

**Conclusions:**

This study proposes VAV3 as a biomarker and a rationale for its use as a signaling target to prevent and/or overcome resistance to endocrine therapy in breast cancer.

## Introduction

Endocrine therapies are the cornerstone of the curative and palliative treatment of ERα-positive breast cancer. However, even patients who initially respond to these therapies may eventually develop resistance. Current knowledge of the molecular mechanisms of acquired resistance to endocrine therapies suggests a model in which crosstalk between ERα and growth factor signaling pathways plays an important role [1-3]. There may also be resistance mechanisms partially or totally independent of growth factor signaling, such as mutations in the *ESR1* gene, which encodes for ERα, that alter ligand and/or coactivator binding [4-6].

Beyond the alterations in growth factor signaling pathways identified to date, the binding plasticity of ERα to chromatin is central in therapeutic resistance and cancer progression [7]. This plasticity is mediated by the interaction of ERα with FOXA1 and, importantly, as a result, a rewired transcriptional program that endorses resistance [8]. In this scenario, however, it is not fully understood which transcriptional outputs—possibly those involved in growth factor signaling pathways—may be critical in the acquisition of the resistant phenotype.

In recent years, different breast cancer cell models have been generated in efforts to decipher the mechanisms of acquired resistance to endocrine therapies [3,9,10]. One popular model was based on the long-term estrogen deprivation (LTED) of the ERα-positive breast cancer cell line MCF7 [11-14]. This model was designed to recapitulate the effects of the therapeutic use of aromatase inhibitors (AIs) in postmenopausal breast cancer [15]. Relevant differences, but also similarities, have been described between the MCF7-LTED model and other cell models of acquired resistance [16,17]. Although this observation raises potential limitations, the results obtained with these models should be evaluated in the corresponding clinical settings. In our present study, in which we start with an analysis of the response of MCF7-LTED cells to different small compounds and then report our testing of predictions in different cohorts of breast cancer patients, we propose that *VAV3*/VAV3 is a key ERα-downstream determinant of the response to endocrine therapies.

## Methods

### Cell culture and viability assays

MCF-7 cells were routinely cultured and maintained in Roswell Park Memorial Institute medium containing 10% fetal bovine serum and 2 mM glutamine. MCF7-LTED cells were established in phenol red-free medium containing 10% dextran-coated, charcoal-stripped serum [17]. All other cell lines used in this study were cultured according to standard protocols [18]. The epidermal growth factor (EGF) (Sigma-Aldrich, St Louis, MO, USA) was used at 10 ng/ml for 5 minutes. Cellular viability was evaluated using standard methylthiazol tetrazolium (MTT)–based assays (Sigma-Aldrich). The results of these assays are expressed relative to vehicle-treated controls and to the original time point.

### Chemical compound screen

MCF7 and MCF7-LTED cells were plated in 384-well microtiter plates, and five compound dilutions (1 nM to 10 μM final concentration) from the Library of Pharmacologically Active Compounds (LOPAC1280) (1,258 compounds; Sigma-Aldrich) were added to the cultures. Cell viability was assessed after 72 hours using MTT-based assays and the EnVision spectrofluorometer (PerkinElmer, Waltham, MA, USA). The screen was performed in triplicate. Data quality was assessed (*Z*′-factor > 0.5 for all screens), and data analysis was performed using the cellHTS2 module in the Screensaver database [19]. The data were normalized between 0 and 1 using positive (1 μM phenylarsene oxide) and negative (0.1% dimethyl sulfoxide (DMSO)) controls. For hit selection, the difference between the normalized percentage inhibition (NPI) in MCF7 and MCF7-LTED cells was calculated by subtraction (ΔNPI = NPI(MCF7-LTED) − NPI(MCF7)), and the differentials were clustered with the MeV software package [20] using the Cluster Affinity Search method with the Euclidean distance metric (threshold of 0.7). Based on the 18 clustered differential profiles, 83% of the compounds (*n* = 1,047) had no differential effect between the cell lines, 1% (*n* = 13) were more selective towards MCF7-LTED cells and 0.5% (*n* = 6) were more selective toward MCF7 cells. The YC-1 compound was purchased from Sigma-Aldrich and from Chemgen Pharma International (custom synthesis order; Calcutta, India), and erlotinib was purchased from Santa Cruz Biotechnology (Santa Cruz, CA, USA).

### cGMP, subcellular fractionation, and Western blotting

The cGMP levels were measured using the Amersham cGMP Direct Biotrak EIA system (GE Healthcare Life Sciences, Pittsburgh, PA, USA). Fractionation was performed with a subcellular protein fraction kit (Thermo Fisher Scientific, Asheville, NC, USA). Cells were lysed in buffer containing 50 mM Tris-HCl pH 8, 0.5% Nonidet P-40, 100 mM NaCl and 0.1 mM ethylenediaminetetraacetic acid, supplemented with protease inhibitor cocktail (Roche Molecular Biochemicals, Indianapolis, IN, USA) and 1 mM NaF. Lysates were clarified twice by centrifugation at 13,000 × *g*, and protein concentration was measured using the Bradford method (Bio-Rad Laboratories, Hercules, CA, USA). Lysates were resolved in SDS-PAGE gels and transferred to Immobilon-P membrane (EMD Millipore, Billerica, MA, USA) or polyvinylidene fluoride membrane (Roche Molecular Biochemicals), and target proteins were identified by detection of horseradish peroxidase–labeled antibody complexes with chemiluminescence using an Amersham ECL Western Blotting Detection Kit (GE Healthcare Life Sciences).

### ERα structural analysis and binding assay

Chains A and C of the RCSB Protein Data Bank (PDB) structure 3OS8 [Swiss-Prot:P03372] were superimposed and used as representative structures of the partially constrained and unconstrained forms, respectively. Hydrogen atoms and protonation states were automatically assigned using the Protonate 3D function of the Molecular Operating Environment (Chemical Computing Group, Montreal, QC, Canada) [21], and the structures were saved in Mol2 file format, which was then used as input for docking analysis in rDock [22]. The cavity was defined as the available space 6 Å around the crystallized ligand. Both WAY6 and YC-1 were docked to each of the conformations in exhaustive sampling mode (100 genetic algorithm runs). The binding mode in chain A (binding mode 1, as previously described [23]) was considered to be responsible for the partial agonist activity, and the binding mode in chain C (binding mode 4, as previously described [23]) caused a shift in the conformation of helices 3 and 11, which displaced helix 12 and resulted in an inactive state. To test the performance of the docking program, WAY6 bound to chain C was cross-docked to chain A, and vice versa. The experimental binding mode of WAY6 was reproduced in both cases, although modes 1 and 4 scored very similarly in chain C, suggesting that these modes can coexist in the unconstrained (inactive) conformation. By contrast, binding mode 4 was clearly disfavored in chain A, indicating that this binding mode is incompatible with the partially constrained (active) conformation. The ERα agonist fluoligand assay was performed by Cerep (Paris, France) using YC-1 final concentrations from 10 to 250 μM.

### Gene expression analyses

RNA samples were extracted using TRIzol reagent (Life Technologies, Carlsbad, CA, USA) and the RNeasy kit (QIAGEN, Valencia, CA, USA), and quality was evaluated in the Agilent 2100 Bioanalyzer (Agilent Technologies, Santa Clara, CA, USA). RNAs were amplified using the Ribo-SPIA system (NuGEN Technologies, San Carlos, CA, USA) and subsequently hybridized on the Human Genome U219 microarray platform (Affymetrix, Santa Clara, CA, USA). The data have been deposited in the Gene Expression Omnibus (GEO) [GSE:38829]. Publicly available whole-genome expression data for 51 breast cancer cell lines were analyzed using the preprocessed and normalized values [18]. The Gene Set Expression Analysis (GSEA) was run using default values for all parameters [24]. Preprocessed and normalized microarray data from breast tumors and tumor response to tamoxifen were taken from the corresponding repositories: the Stanford microarray repository (NKI-295 data set) [25] and the GEO record [GSE:9195], respectively. Cox proportional hazard regression analysis was used to evaluate differences in distant metastasis-free survival according to *VAV3* expression (three microarray probes were treated independently).

### Chromatin immunoprecipitation data analysis

Chromatin immunoprecipitation (ChIP) data were downloaded from the GEO database [GSE:32222] [7] and analyzed using MACS version 2.0.9 software (macs2diff function) [26]. Significance was defined by a *Q*-value <0.01 and using default values for the remaining parameters. Differentially bound genomic regions were annotated to the closest ENSEMBL (hg19) annotated gene using the R-Bioconductor package ChIPpeakAnno [27]. Previously aligned reads were extracted from the sequence read archive [SRP:032421], and sequence counts were normalized to the library size. ERα and nonspecific immunoglobulin control (IgG) ChIP assays were performed as previously described [28,29]. Briefly, the DNA was purified using a phenol-chloroform extraction protocol, the antibodies used were anti-ERα (SC-543 and SC-7207; Santa Cruz Biotechnology) and anti-IgG (ab46540; Abcam, Cambridge, UK), and three independent biological replicates were obtained in all cases. The primers used were site 1: forward 5′-CACTTCCTTTCCTGGTTGGA-3′ and reverse 5′-AGTAAAAGGGGTGCCCTCTC-3′, and site 2: forward 5′- TGTGGTGTTTCCTGTTAGTGG-3′ and reverse 5′- TTGCCAATAACTTAAAGCGTAGG-3′.

### Antibodies and RAC1 activity assay

The antibodies we used were anti-E2F1 (KH95; Santa Cruz Biotechnologies), anti–epidermal growth factor (anti-EGFR) (1005; Santa Cruz Biotechnologies), anti-ERα (SP-1; Abcam), antibody against phosphorylated extracellular signal-regulated protein kinases 1 and 2 (anti-phospho-ERK1/2) (D13.14.4E; Cell Signaling Technology, Danvers, MA, USA), anti-NUP62 (nucleoporin 62 kDa, clone 53; BD Transduction Laboratories, San Jose, CA, USA), anti-PAK1 (2602; Cell Signaling Technology), anti-RAC1 (05-389; EMD Millipore), anti-phospho-serine 235/236 ribosomal S6 (D57.2.2E; Cell Signaling Technology), anti-VAV3 (07-464, Millipore; and 2398, Cell Signaling Technology), anti-phospho-tyrosine 173 VAV3 (anti-pT173 VAV3, ab52938; Abcam) and anti–tubulin α (anti-TUBA) (DM1A *+* DM1B; Abcam). Secondary antibodies for used for immunofluorescence (Alexa Fluor) were obtained from Molecular Probes (Eugene, OR, USA). To measure RAC1 activity, we used the Rac1 G-LISA Activation Assay Biochem Kit (BK128; Cytoskeleton, Denver, CO, USA). The MYC-Vav3 wild-type and oncogenic expression constructs we used have been described previously [30,31].

### Short hairpin RNA assays

For the *ESR1* and *VAV3* expression depletion assays, we used MISSION shRNA (Sigma-Aldrich). The lentiviral packaging, envelope, control and green fluorescent protein (GFP) expression plasmids (psPAX2, pMD2.G, non-hairpin-pLKO.1, scrambled-pLKO.1 and pWPT-GFP) were purchased from Addgene (Cambridge, MA, USA). Production and collection of lentiviral particles were carried out according to a modified Addgene protocol. Initial viral titers >5 × 10^5^/ml were confirmed by Lenti-X GoStix lentivirus testing (Clontech Laboratories, Mountain View, CA, USA), and supernatants were then concentrated by ultracentrifugation or with the Lenti-X Concentrator (Clontech Laboratories) and stored at −80°C. Concentrated viral supernatants were titrated for optimal inhibition of the target.

### Genetic association study

The Institutional Review Board of the Institute of Medical Science (The University of Tokyo) approved the study, and written informed consent was obtained from all patients. A total of 240 patients with primary breast cancer, recruited by the Shikoku-*10 collaborative group (Tokushima Breast Care Clinic, Yamakawa Breast Clinic, Shikoku Cancer Center, Kochi University Hospital and Itoh Surgery and Breast Clinic), Kansai Rosai Hospital, Sapporo Breast Surgical Clinic and Sapporo Medical University Hospital from September 2007 to September 2008, were included in the genome-wide association study (GWAS), and 105 patients recruited by the same centers from October 2008 to January 2010 were included in the replication study. All patients were Japanese women pathologically diagnosed with ERα-positive invasive breast cancer. They received adjuvant tamoxifen monotherapy between 1986 and 2008. The data on primary breast cancer diagnoses or recurrences were confirmed by extraction from the patients’ medical records. Patients without recurrence were censored at the date of the last clinical evaluation. Recurrence-free survival time was defined as the time from surgical treatment to the diagnosis of breast cancer recurrence (locoregional, distant metastasis or contralateral breast events) or death. Patients received tamoxifen 20 mg/day for 5 years. Treatment was stopped at the time of recurrence. Genomic DNA was extracted from peripheral blood or frozen breast tissue using the QIAGEN DNA Extraction Kit. In the GWAS, 240 patients were genotyped using the Illumina Human610-Quad BeadChip array (Illumina, San Diego, CA, USA). Quality control was assured by excluding single-nucleotide polymorphisms (SNPs) with low call rates (<99%) and those with a Hardy–Weinberg equilibrium *P*-value <1.0 × 10^−6^. SNPs with a minor allele frequency <0.01 were also excluded from the analyses. The multiplex PCR-based Invader assay (Third Wave Technologies, Madison, WI, USA) on ABI PRISM 7900HT (Applied Biosystems, Foster City, CA, USA) was used in the replication study. For statistical analysis, recurrence-free survival curves were estimated using the Kaplan–Meier method. The statistical significance of relationships between clinical outcomes and genetic variations was assessed using a logrank test.

### Tumor series and immunohistochemistry

For the Bellvitge Institute for Biomedical Research (IDIBELL, Barcelona, Spain) cohort, the IDIBELL Ethics Committee approved the study and written informed consent was obtained from all patients. Twenty-nine patients treated with primary endocrine therapy before surgical excision of breast tumors were chosen from the clinical database activity of the Catalan Institute of Oncology (ICO) Breast Cancer Unit. All patients were postmenopausal and diagnosed with ERα -positive and HER2-negative breast cancer. The patients received treatment with either an ERα antagonist (tamoxifen or toremifene) or an aromatase inhibitor (letrozole or exemestane). Patients received therapy until a maximum response was achieved (range, 4 to 27 months), unless tumor progression was observed during a twice-monthly radiological and clinical assessment. After endocrine therapy was completed, full tumor excision was performed by either lumpectomy or radical mastectomy. Response was defined as the percentage of fibrosis and other patterns of pathological response attributable to tumor reduction at surgery. Tissue was obtained at surgery or biopsy, fixed in buffered formalin and processed for use in paraffin-embedded sections. A Stockholm cohort was analyzed in the Swedish study, which consisted of postmenopausal breast cancer patients enrolled in a randomized adjuvant trial between November 1976 and April 1990. The study design and long-term follow-up data were previously reported in detail [32]. Ethical approval for the Swedish study was obtained from the Karolinska Institute Ethics Council. Immunohistochemistry was performed using the heat-mediated antigen retrieval method with citrate buffer. The VAV3 polyclonal antibody used for immunohistochemistry has been described previously [30]. Scoring of the immunohistochemical results was performed in a blind and independent manner by two pathologists.

## Results

### A chemical compound screen identifies YC-1 as reducing viability of cellular models of acquired resistance

**Figure 1:**
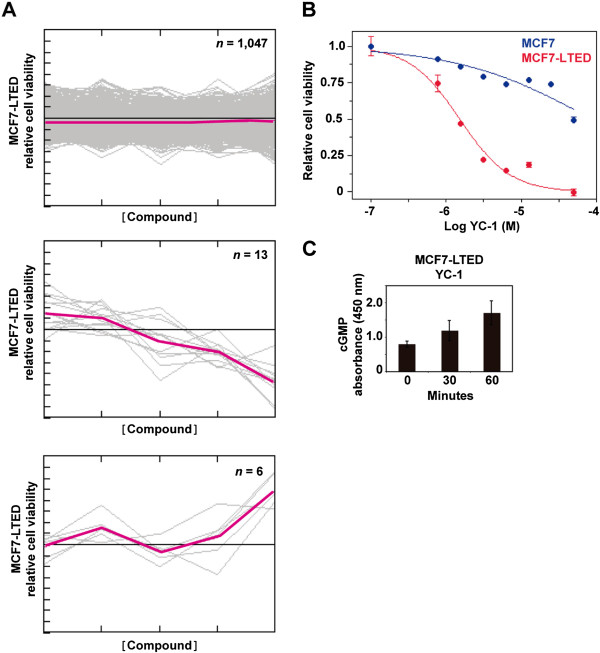
**A chemical compound screen identifies an activator of soluble guanylyl cyclase as reducing the viability of long-term estrogen-deprived MCF7 cells. (A)** Compounds with no differential effect (top panel), with an inhibitory effect on the long-term estrogen deprivation (LTED) of MCF7 cells (MCF7-LTED cells) relative to MCF7 cells (middle panel) and with an inhibitory effect on MCF7 relative to MCF7-LTED cells (bottom panel). The *y*-axis indicates relative viability of MCF7-LTED cells, and the *x*-axis indicates increasing concentrations of the compounds. Colored lines indicate average values. **(B)** Corroboration of the inhibitory effect of YC-1 on MCF7-LTED cells. **(C)** Time-dependent increase of cGMP in MCF-LTED cells exposed to YC-1.

Acquired resistance to aromatase inhibitors in postmenopausal women can be modeled in MCF7-LTED cells [17]. Using this model, we carried out a cell-based chemical compound screen out to identify potential therapeutic strategies that could prevent and/or overcome resistance. More than 1,200 compounds were assessed for their differential effect on the viability of MCF7-LTED cells (as defined by MTT-based assays) relative to the parental MCF7 cells. Thirteen compounds showed higher relative inhibition in MCF7-LTED cells (Figure 1A and Additional file 1: Table S1). Subsequent validation using independent cell cultures and compound solutions identified YC-1 (3-(5′-hydroxymethyl-2′-furyl)-1-benzylindazole) as being the most effective, with a 27-fold difference in the half-maximal inhibitory concentration (IC_50_) was revealed between MCF7-LTED and MCF7 cells (4.9 μM and 131 μM, respectively) (Figure 1B).

YC-1 is a direct activator of soluble guanylyl cyclase (sGC) Thus, increased levels of cGMP were observed in cell cultures exposed to this compound (Figure 1C). Next, the effect of YC-1 on a collection of breast cancer cell lines was examined. IC_50_ values <10 μM were obtained for several cell lines (Additional file 2: Table S2), including MCF7-LCC9 and MCF7-LY2, which correspond to models of acquired resistance to fulvestrant and to the raloxifene analogue LY-117018, respectively. These cell lines also showed cross-resistance to tamoxifen [33,34].

Intriguingly, an activator of sGC derived from the structural development of YC-1, BAY 41-2272, displayed a lower differential inhibitory effect (Additional file 3: Figure S1A). In addition, assessment of another sGC activator, A-350619, and complementary evaluation of an inhibitor of phosphodiesterase activity did not reveal the expected differences (Additional file 3: Figure S1B). Although YC-1 has been used extensively in cancer research, including preclinical studies in breast cancer [35], it is unclear whether a direct target beyond sGC exists.

### YC-1 binds to estrogen receptor α

**Figure 2:**
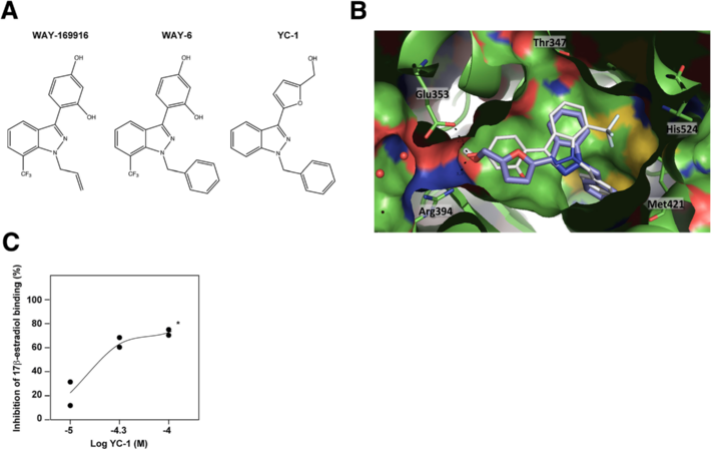
**YC-1 binds to estrogen receptor α. (A)** Chemical structures of WAY-169916, WAY6 and YC-1. **(B)** Predicted binding mode of YC-1 (purple) in the partially constrained conformation of estrogen receptor α (ERα) (chain A, Protein Data Bank code 3OS8 [Swiss-Prot:P03372]). The binding mode of WAY6 (white sticks) is shown as a reference. **(C)** The results of the ERα agonist fluoligand assay using YC-1 are shown, along with the concentration–inhibition curve with duplicates. *YC-1 was not completely soluble at concentrations >100 μM.

To investigate novel molecular targets of YC-1, the chemical structure of YC-1 was used to query the ChEMBL [36] and BindingDB [37] databases for similar compounds with reported biological activity. Strikingly, WAY-169916, which has been shown to bind ERα [38], and a series of related compounds [23,39] were retrieved at a 60% similarity cutoff value. WAY-169916 is an unusual ERα ligand: It is able to bind ERα, leading to its constrained or unconstrained conformation (responsible for partial agonist activity, binding mode 1, or for an antagonist effect, binding mode 4, respectively) [23]. The relative preferences for these ERα conformations explain the graded activities across the compound series [23]. Thus, compound 6 (hereinafter referred to as WAY6) was the WAY-169916 analogue most similar to YC-1 (Figure 2A), which was found to lead preferentially to the unconstrained conformation [23].

Molecular docking was used to examine the potential binding mode of YC-1 to ERα. The predicted mode was very similar to binding mode 1 of WAY6 when docked in both the partially constrained (Figure 2B) and unconstrained (Additional file 4: Figure S2A) conformations. Although a binding mode similar to binding mode 4 was also found to be possible in the latter conformation (Additional file 4: Figure S2B), it had a lower score. As shown in Figure 2B, the binding mode of YC-1 was almost perfectly aligned with WAY6 and maintained the main molecular interactions with ERα, which comprised van der Waals contacts with the lipophilic cavity and a double hydrogen bond with Glu353 and Arg394. The absence of the trifluoromethyl group, which is engaged in a weak hydrogen bond with His524, could cause some loss of potency, but this group was not essential for the biological activity in the WAY-169916 series [38].

The MCF7-LTED model was previously shown to be less sensitive to fulvestrant than the parental MCF7 [17], and this difference appeared to be coherent with the described differential ERα binding mode of fulvestrant relative to WAY-169916 [23]. Next, to validate the binding prediction between YC-1 and ERα, we performed an agonist fluoligand assay, which showed the competition with fluorescein-labeled estradiol. The results of this assay revealed YC-1 IC_50_ and K_i_ values of 33 μM and 26 μM, respectively (Figure 2C), which are in agreement with the inhibitory effects observed in the cell lines (Additional file 2: Table S2). Intriguingly, two of the cell lines that showed relative inhibition by YC-1 (AU565 and SKBR3) are generally considered ERα-negative [18]. Thus, the combined targeting of at least sGC and ERα would make it difficult to interpret the phenotypic consequences of therapy based on YC-1. Consequently, the specific molecular perturbations mediated by YC-1 should be identified.

### Molecular perturbations mediated by YC-1

Having defined breast cancer cell lines with relatively higher sensitivity to YC-1, we evaluated the existence of a common molecular signature among these lines. The GSEA [24] tool was used to examine gene set expression differences between cell lines of “high” and “low” sensitivity (defined by an IC_50_ threshold of 10 μM) (Additional file 2: Table S2). The cell lines with higher sensitivity to YC-1 had overexpression of cell cycle pathway genes, whereas the less sensitive cell lines cells showed overexpression of ribosome pathway genes, among others (Additional file 5: Figure S3 and Additional file 6: Table S3). These results are consistent with the increased dependence of the cell cycle and proliferation highlighted in endocrine therapy resistance in previous studies [40].

To examine the potentially selective YC-1 mechanism of action in models of acquired resistance, the levels and subcellular localization of ERα were examined. Although both were altered by YC-1 treatment, no substantial differences were observed between MCF7 and MCF7-LTED cells (Additional file 7: Figure S4). Subsequently, whole-genome expression data were obtained for both cell lines in basal and YC-1 exposure conditions. Consistent with the results described above, expression of the ribosome pathway was clearly differentiated between MCF7 and MCF7-LTED cells in basal conditions and with exposure to YC-1 (Additional file 8: Figures S5A and S5B and Additional file 9: Table S4). Exposure to YC-1 led to a significant alteration of the cell cycle pathway in both settings (Additional file 8: Figure S5B). Accordingly, targets of a central positive regulator of the cell cycle, E2F1, were revealed to be significantly underexpressed with exposure to YC-1 (Additional file 10: Table S5). Protein analysis revealed a larger relative decrease in the expression of this transcription factor in MCF7-LTED cells exposed to YC-1 (Additional file 8: Figure S5C). Together, these results indicate that YC-1 may reduce the potential of cell proliferation in such a way that MCF7-LTED cells are relatively more sensitive.

**Figure 3:**
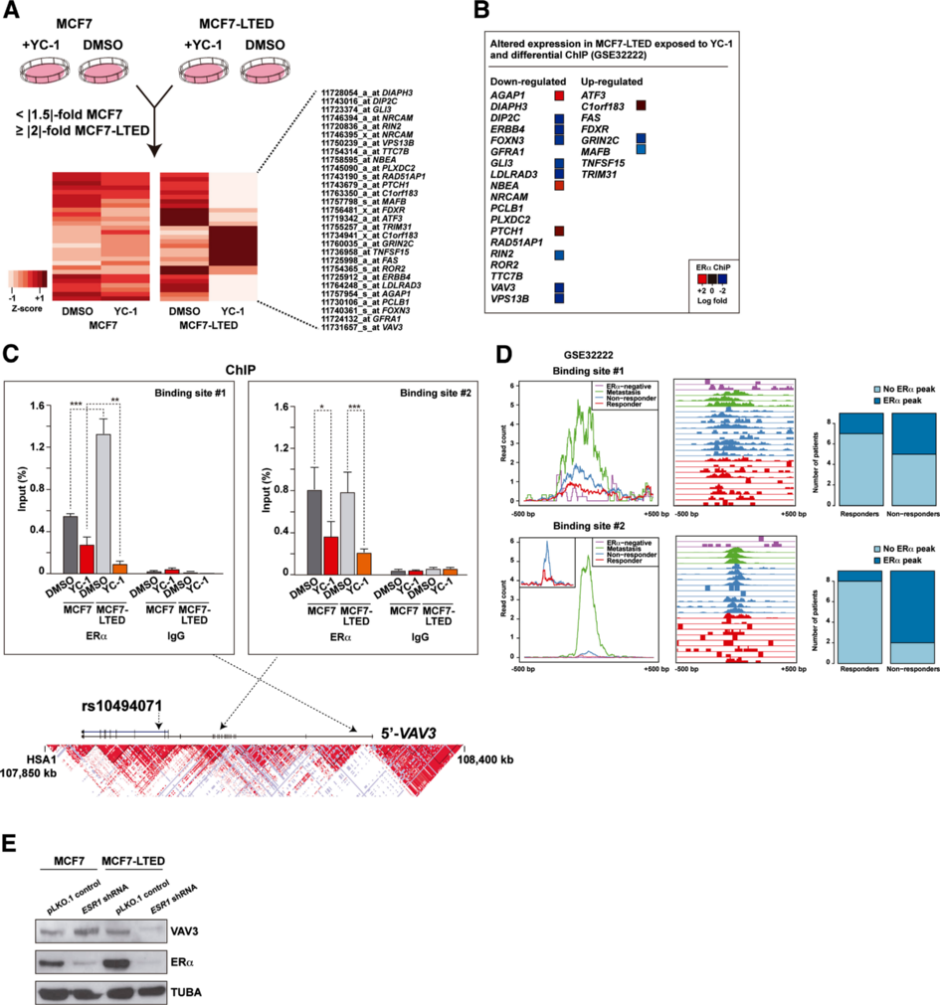
**Genes specifically perturbed by YC-1 in long-term estrogen deprivation of MC7 cells and their link to the response to endocrine therapy. (A)** Genes whose expression change differentiates the effect of YC-1 between long-term estrogen deprivation of MCF7 (MCF7-LTED) cells and MCF7 cells. The bottom heatmap shows the normalized expression differences for the probes and genes that passed the defined thresholds. DMSO, Dimethyl sulfoxide. **(B)** Logarithmic fold changes between the responder and nonresponder breast tumors for the genes (Gene Expression Omnibus data set [GSE:32222]) shown in **(A)** determined by chromatin immunoprecipitation (ChIP) assay. **(C)** ChIP assay results for estrogen receptor α (ERα) and immunoglobulin G (IgG) at two sites in the *VAV3* locus, both for MCF7 and MCF7-LTED cells with or without exposure to YC-1 (significant differences are indicated by asterisks: *P < 0.05, **P < 0.01, ***P < 0.001). The bottom graph shows the genomic locus with the linkage disequilibrium structure in Japanese individuals found in HapMap and the relative position of the variant rs10494071 (presented below). **(D)** Detailed analysis of the Gene Expression Omnibus [GSE:32222] data set for the two sites depicted above. Left panels show the normalized average intensity of ERα binding ±500 bp around the sites in different sample sets as depicted in the insets. Middle panels show relative ERα binding in the above sites across 23 breast cancer samples. Right panels are graphs showing the number of cases with or without an ERα binding event (peak) in nonresponders and responders. Top right panels show that 44% of the nonresponders had ERα binding, whereas only two (22%) of nine responders had binding. Bottom right panels show that 78% of the nonresponders had ERα binding, whereas only one (11%) of nine responders had binding. **(E)** Short hairpin RNA (shRNA)–mediated depletion of ERα led to a decrease in VAV3 levels in MCF7-LTED cells, but not in MCF7 cells.

Having observed pathway differences, we aimed to identify the largest gene expression differences between MCF7 and MCF7-LTED cells exposed to YC-1. Thus, we defined a twofold or greater change in MCF7-LTED cells (between basal and YC-1 conditions), and a 1.5-fold or greater expression change in MCF7 cells. In this analysis, we identified 19 and 8 genes, respectively, that were down- and upregulated in MCF7-LTED cells exposed to YC-1 (Figure 3A). Consistent with the binding of YC-1 to ERα, many of these perturbed genes corresponded to loci that are differentially regulated by ERα in endocrine therapy resistance. Analysis of ChIP data of responsive and nonresponsive breast tumors [7] revealed significant differential ERα binding at several of these loci, with 10 of 27 showing increased binding in the nonresponsive setting (Figure 3B). From among this set, *VAV3* was further included in a 271-gene list associated with poor clinical outcome [7]. Following on from these observations, we performed ERα ChIP assays using extracts of MCF7 and MCF7-LTED cells in basal (DMSO) or YC-1-exposed conditions. By this method, we found two *VAV3* sites with significant binding of ERα relative to the nonspecific immunoglobulin control (Figure 3C). In addition, both sites showed ERα sensitivity (that is, lower binding) with exposure to YC-1, and one site (binding site 1) had significantly more binding (2.4-fold) in MCF7-LTED cells than in MCF7 cells (Figure 3C). Similarly, specific analysis of these sites in the original breast cancer data set [7] showed substantial ERα binding in nonresponder and metastasis cases (Figure 3D). Consistent with these observations, and among the potential ERα downstream effectors identified above, *VAV3* showed the highest expression associated with *ESR1* in breast tumors [25] (mutual information = 0.23, *P* < 0.001). Moreover, shRNA-mediated depletion of ERα revealed a decrease of VAV3 in MCF7-LTED cells, but not in parental MCF7 cells (Figure 3E). Collectively, these results indicate that VAV3 function may be critical in endocrine therapy resistance governed by ERα transcriptional regulatory plasticity.

### VAV3 is perturbed by YC-1 and determines acquired resistance

**Figure 4:**
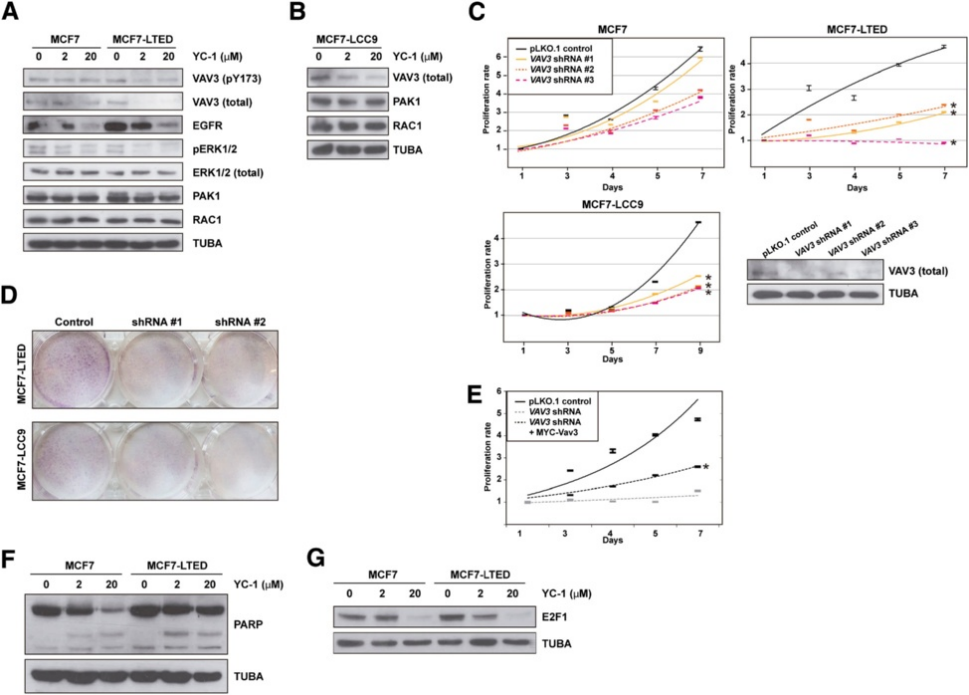
**Study of VAV3 in models of acquired resistance to endocrine therapies. (A)** Western blot analysis results for VAV3 (pT173 and total, top panels), signaling components and control tubulin α (TUBA) from MCF7 cells and long-term estrogen deprivation of MCF7 (MCF7-LTED) cells in basal and YC-1 exposure conditions. pERK, phosphorylated extracellular signal-regulated protein kinase. **(B)** Western blot analysis results for VAV3, PAK1 and RAC1, as well as control TUBA, in MCF7-LCC9 cell extracts from basal and YC-1 exposure conditions. **(C)** Short hairpin RNA (shRNA)–mediated depletion of VAV3 reduces the viability, in methylthiazol tetrazolium (MTT)–based assays) of MCF-LTED and MCF7-LCC9 cells relative to parental MCF7. The asterisks correspond to significant differences (*P* < 0.05) in the viability rate (slope of the trends (shown), including three replicas, and relative to the control pLKO.1). The bottom right panel shows the results of short hairpin RNA (shRNA)–mediated depletion of VAV3 relative to the negative control assay. **(D)** shRNA-mediated depletion of VAV3 reduces the viability (clonogenic assays) of MCF-LTED and MCF7-LCC9 cells. **(E)** Reconstitution with MYC-Vav3 partially recovers viability of MCF-LTED cells. The asterisk corresponds to a significant difference (*P* < 0.05) relative to shRNA-mediated depletion of VAV3. **(F)** No substantial differences in poly(ADP-ribose) (PARP) cleavage were observed between MCF7 and MCF7-LTED cells exposed to YC-1. **(G)** Top panel, reduction of E2F1 expression in MCF7 and MCF7-LTED cells exposed to YC-1; MCF7-LTED, but not the parental MCF7, show a reduction at 2 μM YC-1. Bottom panel, control TUBA.

Consistent with the observations described above, total and pY173 VAV3 (whose phosphorylation regulates activity) [31,41] decreased in MCF7-LTED cells, but not in MCF7 cells, exposed to YC-1 (Figure 4A). According to the position of VAV3 in its canonical signaling pathway, EGFR levels were decreased in both MCF7 and MCF7-LTED cells exposed to YC-1, but ERK1/2 phosphorylation was decreased only in MCF7-LTED cells exposed to YC-1 (Figure 4A). In addition, PAK1 and RAC1 levels were not altered under these conditions (Figure 4A). Similarly to MCF7-LTED, MCF7-LCC9 cells exposed to YC-1 showed loss of expression of VAV3, but not of PAK1 or RAC1 (Figure 4B). Nonetheless, depletion of VAV3 reduced RAC1 activity in both MCF7 and MCF7-LTED cells, and this alteration was recovered through reconstitution using a shRNA-resistant MYC-Vav3 expression construct (Additional file 11: Figure S6).

Next, lentivirus-mediated transduction of shRNAs directed against expression of *VAV3* significantly reduced the viability of MCF7-LTED and MCF7-LCC9 cells relative to MCF7 cells (*P* < 0.05) (Figure 4C). A clonogenic assay also indicated relative loss of viability of MCF7-LTED cells, and, to a lesser extent, MCF7-LCC9 cells, with shRNA-mediated depletion of VAV3. Differences relative to MCF7 cells were <0.8-fold (Figure 4D). Reconstitution with MYC-Vav3 significantly recovered proliferation in MCF7-LTED cells, although not to the level of the shRNA control assay (Figure 4E), which might have been due to Vav3 overexpression (Additional file 11: Figure S6) and/or to specific roles of splicing variants. Reconstitution with Vav1 or Vav2 could not be assessed, as the overexpression of the murine counterparts caused cell death (data not shown). Analysis of poly(ADP-ribose) cleavage did not reveal substantial differences among the cell lines (Figure 4F), which further indicates that YC-1 primarily inhibits cell proliferation. Thus, a reduction in E2F1 was observed in MCF7-LTED cells exposed to 2 μM YC-1 (Figure 4G). These results are also consistent with those of VAV3 depletion in prostate cancer cells [42].

### *VAV3*/VAV3 association with clinical outcome

**Figure 5:**
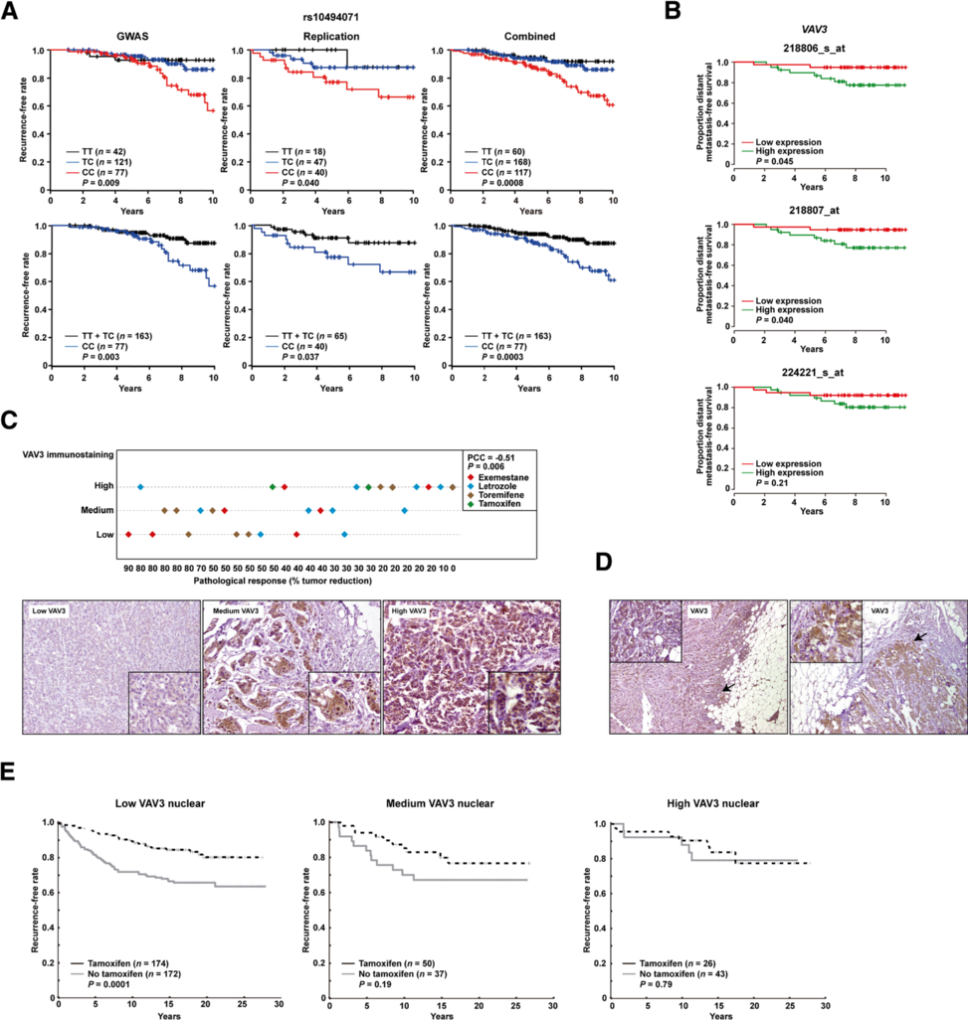
**Association between *VAV3*/VAV3 and clinical response to endocrine therapies. (A)** Association between rs10494071 and the response to tamoxifen in Japanese patients. The Kaplan–Meier curves show the recurrence-free survival rate over time (years) and between patients stratified according to the three possible rs10494071 genotypes (TT, TC and CC). The logrank test *P*-values are shown. **(B)** Association between *VAV3* tumor expression and response to tamoxifen (Gene Expression Omnibus data set [GSE:9195]). Graphs show the proportion of patients with metastasis-free survival over time (years) and stratified according to high (above the median) or low (below the median) *VAV3* expression in breast tumors. The results shown are for three *VAV3* microarray probes. Logrank *P*-values are shown. **(C)** Association between VAV3 tumor expression and the pathological response to endocrine therapies. Top panel: Graph depicting the correlation between VAV3 immunostaining score and pathological response (percentage of tumor reduction posttreatment). Bottom panels: Representative examples of the three immunostaining scores. Insets: Cells with nuclear and cytoplasmic positivity. PCC, Pearson’s correlation coefficient. **(D)** Examples of increased VAV3 staining at the invasive tumor front. **(E)** Graphs showing the Kaplan–Meier curves for patients who did or did not receive tamoxifen in the Swedish study. Panels from left to right show the results for patients whose tumors revealed low, medium or high nuclear VAV staining. The logrank test *P*-values are shown.

Having identified VAV3 as a determinant of acquired resistance in cellular models, we next assessed its relevance in the clinical scenario. By examining the results of a Japanese GWAS regarding response to tamoxifen [43], we identified 20 SNPs in *VAV3* that are associated with clinical outcomes (logrank *P*-values <0.05) (Additional file 12: Table S6). In a subsequent assessment of an independent patient series, the associations in several SNPs were replicated. Of the variants analyzed, rs10494071 showed the strongest association in the combined analysis (*P* = 8.4 × 10^−4^) (Figure 5A). The rs10494071 variant is located within *VAV3* intron 19 (Figure 3C) and may represent an expression quantitative trait locus. In a study of monocytes [44], the minor allele was associated with lower expression levels of *VAV3* (*P* = 2.2 × 10^−11^).

An association between the rs10494071 minor allele, which in turn was associated with a better tamoxifen response (Figure 5A), and lower germline expression of *VAV3* seemed to be consistent with mediation of resistance by this signaling component. Next, we analyzed an expression data set from ERα-positive breast cancer patients treated with tamoxifen [45]. The results of this analysis also suggest that low *VAV3* expression might be associated with better outcomes (logrank *P*-values <0.05 for two probes) (Figure 5B).

Complementarily to the germline association study, we assessed a series of 29 breast tumors, which had been collected by biopsy after endocrine therapy, for VAV3 expression by immunohistochemistry. A negative correlation (Pearson’s correlation coefficient = −0.51, *P* = 0.006) was revealed between the scores of VAV3 staining (low, medium or high) and the pathological response to therapy (that is, tumor reduction) (Figure 5C). These 29 cases included a variety of endocrine therapies, but no bias with respect to therapy type was apparent. Moreover, consistent with the role of VAV3 in promoting breast cancer progression [30], comparatively higher staining was observed at the tumor fronts (Figure 5D). In addition, higher staining scores could be linked to nuclear positivity (insets in Figures 5C and 5D), and, intriguingly, this localization has previously been shown to be necessary for the function of the androgen receptor in prostate cancer [46].

To further assess the above-described immunohistochemical association, we performed an independent tumor tissue microarray analysis with detailed molecular, histopathological and clinical information [32,47-49]. The results of this study revealed a significant association between the benefit of tamoxifen therapy and low nuclear VAV3 staining. Conversely, high nuclear VAV3 was not associated with tamoxifen benefit (Figure 5E). In addition, nuclear VAV3 was found to be positively correlated with markers of poor therapy response, particularly phospho-Ser305 ERα and nuclear phospho-Ser473 AKT (*P*-values <0.01) (Table 1) [48]. These correlations, and those between cytoplasmic VAV3 and tumor size and grade, as well as ERα/PR status, were analogous to those previously observed for nuclear and cytoplasmic PAK1 [49] (Table 1). The interpretation of the negative and positive correlations, respectively, of phospho-Ser65 4EBP1 and nuclear S6K2 [47] with nuclear VAV3 may be more complex; indeed, we observed a modest correlation between cytoplasmic VAV3 and phospho-Ser2448 mammalian target of rapamycin (mTOR) (*P* = 0.034). Together, these data reinforce the link between the VAV3 signaling axis and resistance to endocrine therapy.

**Table 1:** **VAV3 nuclear and cytoplasmic expression in relation to other tumor markers assessed by the Spearman’s rank correlation**^**a**^

	**Nuclear VAV3, *n *** **(%)**				**Cytoplasmic VAV3, *n *** **(%)**			
**Score**	**–**	**1+**	**2+**	**3+**	**–**	**1+**	**2+**	**3+**
All tumors	607 (85.9)	3 (0.4)	43 (6.1)	54 (7.6)	229 (32.4)	154 (21.8)	215 (30.4)	109 (15.4)
Tumor size (>20 mm vs. ≤20 mm)	*R*_s_ = −0.04, *P* = 0.30				*R*_s_ = 0.13, *P* = 0.0009			
Tumor grade (1, 2 or 3)	*R*_s_ = −0.09, *P* = 0.026				*R*_s_ = 0.16, *P* = 0.00007			
ERα (>10% vs. ≤10%)	*R*_s_ = 0.05, *P* = 0.20				*R*_s_ = −0.12, *P* = 0.002			
PR (>10% vs. ≤10%)	*R*_s_ = 0.06, *P* = 0.14				*R*_s_ = −0.15, *P* = 0.0002			
HER2 status (positive vs. negative)	*R*_s_ = 0.00, *P* = 0.99				*R*_s_ = 0.05, *P* = 0.16			
Phospho-Ser167 ERα (%)	*R*_s_ = 0.12, *P* = 0.002				*R*_s_ = −0.11, *P* = 0.003			
Phospho-Ser305 ERα (%)	*R*_s_ = 0.11, *P* = 0.006				*R*_s_ = −0.09, *P* = 0.016			
PAK1 (cytoplasm 0 to 3 positivity)	*R*_s_ = −0.07, *P* = 0.077				*R*_s_ = 0.12, *P* = 0.003			
Phospho-Ser473 AKT (nuclear %)	*R*_s_ = 0.18, *P* < 0.00001				*R*_s_ = −0.20, *P* < 0.00001			
Phospho-Ser2448 mTOR (high vs. low)	*R*_s_ = 0.06, *P* = 0.11				*R*_s_ = −0.08, *P* = 0.034			
Phospho-Ser65 4EBP1 (cytoplasm 0 to 2 positivity)	*R*_s_ = −0.15, *P* = 0.0001				*R*_s_ = 0.19, *P* < 0.00001			
S6K2 (nuclear %)	*R*_s_ = 0.21, *P* < 0.00001				*R*_s_ = −0.26, *P* < 0.00001			

^a^ERα, Estrogen receptor α; mTOR, mammalian target of rapamycin; PR, Progesterone receptor. *P* < 0.05 values are statistically significant.

### Therapeutic strategy based on VAV3 evidence

**Figure 6:**
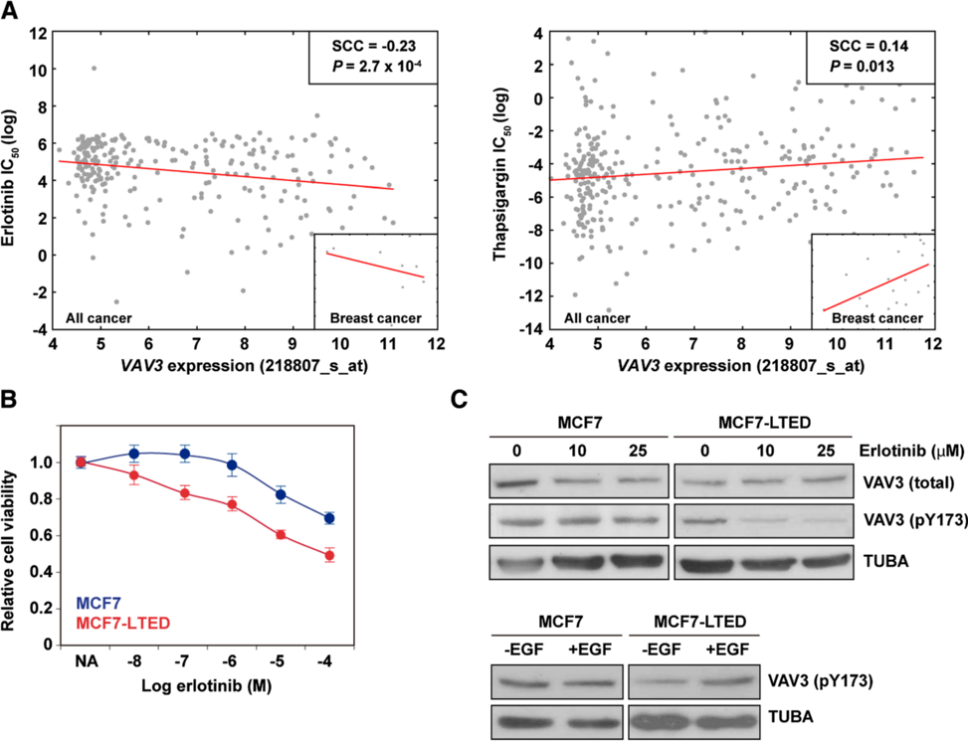
**Correlation analysis between *VAV3 *expression and compounds: half-maximal inhibitory concentration identifies erlotinib as a potential therapeutic compound. (A)** Graph showing the correlation between *VAV3* expression (two probes showed similar results, depicted for 218807_s_at) and erlotinib (left panel) or thapsigargin (right panel) logarithmic half-maximal inhibitory concentration (IC_50_) values across all cancer cell lines. Spearman’s correlation coefficient (SCC) and the corresponding *P*-values are shown. Red lines indicate trends, and insets show results for breast cancer cell lines only. **(B)** Graph showing the inhibitory effect of erlotinib on long-term estrogen-deprived MCF7 (MCF7-LTED) cells relative to parental MCF7 cells. **(C)** Top panels, Western blot analysis results for VAV3 (total), pT173 VAV3 and control tubulin α (TUBA) from MCF7 and MCF7-LTED cells in basal and erlotinib exposure conditions. Bottom panels, Western blot analysis results for pT173 VAV3 and control TUBA from MCF7 and MCF7-LTED cells with or without epidermal growth factor (EGF).

Therapy based on YC-1 should be discouraged because of its multiple targets. In addition, to date, no compounds that specifically target VAV proteins have been identified. Having identified a critical role for VAV3, we hypothesized that compounds whose IC_50_ value is inversely correlated with *VAV3* expression might represent promising therapeutic strategies for the endocrine therapy–resistant setting. To test this hypothesis, we analyzed data from the Genomics of Drug Sensitivity in Cancer project [50]. In this analysis, we found that the strongest positive and negative IC_50_ correlations with *VAV3* expression across all cancer cell lines were for thapsigargin and erlotinib, respectively (Figure 6A). These correlations appeared robust in the analysis of breast cancer only (Figure 6A, insets). Notably, the finding that *VAV3* expression opposes the effect of thapsigargin is congruent with those of previous studies of VAV proteins [51,52]. Conversely, erlotinib inhibits EGFR, which has been extensively linked to endocrine therapy resistance [1,53]. Importantly, VAV3 functions downstream of receptor protein tyrosine kinases, which include EGFR [54]. In accordance with these observations, exposure to erlotinib significantly reduced the viability of MCF7-LTED relative to MCF7 cells (Figure 6B). VAV3 expression was not reduced by exposure to erlotinib (contrary to exposure to YC-1), but we observed a partial reduction in pY173 VAV3 in MCF7-LTED cells (Figure 6C, top panels). Accordingly, exposure to EGF increased pY173 VAV3 in this setting (Figure 6C, bottom panels). Collectively, these results further endorse a critical role for VAV3 in endocrine therapy resistance.

## Discussion

The results of this study suggest that VAV3 function mediates the response to endocrine therapies in breast cancer and, as a result, the acquisition of resistance. In this context, VAV3 might be a key effector whose expression is differentially regulated by ERα [7]. Thus, the expression regulation of VAV3 would be relatively more dependent on ERα in the endocrine therapy–resistant setting. Conversely, in previous studies, researchers have proposed that VAV3 is an activator of ERα [55,56]. These observations could indicate the existence of a feedback mechanism that would ultimately regulate growth factor signaling. Indeed, VAV3 has been shown to activate receptor protein tyrosine kinases and RAC1 [54-56], and an inhibitor of this protein can decrease both estrogen-induced cell proliferation and MCF7-tamoxifen-resistant cell growth [56]. Notably, authors of an independent report identified VAV3 as a marker for posttreatment recurrence of prostate cancer [57]. Together with our analysis of VAV3 in breast tumors, these observations further endorse the link between the VAV3-RAC1-PAK1 signaling axis and resistance to endocrine therapies. Nevertheless, analysis of differential gene expression by exposure to YC-1 may point to complementary mediators of endocrine therapy resistance. Activation of ERBB4 has previously been linked to this setting [58-60], and two other identified perturbations (*GLI3* and *PTCH1*) belong to the Hedgehog signaling pathway, which has been highlighted as a possible therapeutic target in this setting [61]. Whether these proteins act functionally in concert with VAV3 or whether they represent necessary alterations in different biological processes or pathways remains to be determined.

The association between genetic variation in *VAV3* and the response to tamoxifen could allow the stratification of patients according to potential clinical benefit. However, this association should be replicated in independent studies with larger samples. The rs10494071 minor allele has a relatively high frequency in the Japanese population, but is rare in individuals of European ancestry (45% and 5%, respectively, according to HapMap data). This is also the case with a variant in linkage disequilibrium with rs10494071 (data not shown). These observations indicate that an attempt to replicate the association in a non-Japanese population will require dense genotyping at the specific locus.

Although the results of the genetic association should be replicated, they are consistent with the anticipated functional role of VAV3 and with the observations made in gene expression analyses. In our present study, we identified an association between the rs10494071 minor allele and better tamoxifen response, and, in turn, we found in our analysis of a tumor data set that low *VAV3* expression correlates with better tamoxifen response [45]. Additionally, these observations seem to be coherent with the role of the rs10494071 variant as an expression quantitative trait locus for *VAV3*, with the minor allele being associated with significantly lower gene expression in monocytes [44]. Importantly, in a previous study in which the researchers identified VAV3 as a marker for posttreatment recurrence of prostate cancer, the association was in the same direction [57]. Moreover, these results are consistent with, and the conclusions further endorsed by, the associations revealed for nuclear VAV3 and tamoxifen therapy response, as well as the observed correlations between the expression of VAV3 and known tumor markers linked to therapy response. However, further work is required to elucidate the functional difference between nuclear and cytoplasmic VAV3, which is reminiscent of the results for PAK1 [49] and could be linked to the activation of the androgen receptor, as previously shown in prostate cancer [46,62].

It has been firmly established that growth factor signaling influences the response to endocrine therapies and, consequently, the acquisition of resistance. Among other evidence, overexpression of growth factor receptors, including EGFR, has been associated with decreased sensitivity to endocrine therapy and poorer prognosis [63]. Akin to this observation, other researchers have reported that cell models of endocrine therapy resistance overexpress several growth factor receptors, also including EGFR [17]. In turn, these observations have led to the design of clinical trials to assess the target inhibition of the receptors [64]. In this scenario, the analysis of VAV3 expression and/or function could potentially help to identify patients that may benefit from therapies aimed at preventing and/or overcoming endocrine therapy resistance.

## Conclusions

In this study, we have identified VAV3 as a critical mediator of endocrine therapy resistance in breast cancer downstream of ERα and growth factor receptor signaling. The expression of VAV3 may be specifically regulated by ERα in the endocrine therapy–resistant setting. The results of our genetic and immunohistochemical studies indicate that *VAV3*/VAV3 represents a promising biomarker for predicting the response to endocrine therapies. Despite the lack of targeted therapies for VAV proteins, inhibition of EGFR signaling could potentially prevent and/or overcome endocrine therapy resistance mediated by VAV3.

## Abbreviations

ChIP: Chromatin immunoprecipitation; EGFR: Epidermal growth factor receptor; ERα: Estrogen receptor α; GSEA: Gene set expression analysis; GWAS: Genome-wide association study; IC_50_: Half-maximal inhibitory concentration; LTED: Long-term estrogen-deprived; MTT: Methylthiazol tetrazolium; PDB: Protein Data Bank; sGC: Soluble guanylyl cyclase; shRNA: Short hairpin RNA; SNP: Single-nucleotide polymorphism.

## Competing interests

The authors declare that they have no competing interests.

## Authors’ contributions

HA, AU and MAP conceived the project and coordinated the experiments and data analyses. HA, PH and RLB performed the compound screen. JSM, NB and MAP carried out the microarray data analyses. XB performed the protein structure analyses. AI, EN and WZ performed the ChIP data analysis. LC, HA, MAP and LDC performed the targeted ChIP assays. HA, NG, GM and LGB performed the cellular and molecular studies. HA and LC performed the *ESR1* shRNA-based assays. KK, TM, YN and HZ performed the genetic association study. NG, FC, MTS, ARV, MG, AIE, ABRP and XRB performed the tumor and immunohistochemical studies. JBo, EK, GPT, TF, DCS and OS performed the analyses of the Swedish breast cancer study. HA, JSM, MV, ME and MAP contributed the cell lines and performed the erlotinib analysis. RGM, MPHMJ, JBr, AF, JBa, RC, KLB, KEC, JAK and AV contributed the reagents and to the experimental design. MAP drafted the manuscript. All authors read and approved the final manuscript.

## Supplementary Material

Additional file 1: Table S1 Results from the chemical compound screen. Click here for file

Additional file 2: Table S2 Values of YC-1 IC_50_ (μM) in breast cancer cell lines. Click here for file

Additional file 3: Figure S1 Assessment of the activation of sGC in the viability inhibition of MCF7-LTED cells. **(A)** BAY 41-2272 shows an effect, but less than that of YC-1. **(B)** A-350619 (activator of sGC) and sulindac sulfide (inhibitor of phosphodiesterase) do not show the predicted effects in MCF7-LTED cells. In fact, the contrary is observed; A-350619 appears to be more effective in MCF7 cells. Click here for file

Additional file 4: Figure S2 Study of the binding mode of YC-1 to ERα. **(A)** Predicted binding mode of YC-1 (purple) in the unconstrained conformation of ERα (chain C, PDB code 3OS8). The binding mode of WAY6 (white sticks) is shown as reference. **(B)** Docking pose of YC-1 (purple) in the unconstrained conformation of ERα (chain C, PDB code 3OS8) resembling the experimentally observed structure. This binding mode is three score units worse than the one shown above. The binding mode of WAY6 (white sticks) is shown as reference. Click here for file

Additional file 5: Figure S3 Signaling pathways differentially expressed between breast cancer cell lines “sensitive” and “insensitive” to YC-1 exposure (defined by the IC_50_ 10 μM threshold). **(A)** High expression of the cell cycle pathway shows significant association (false discovery rate <5%) with YC-1 sensitivity. Pathway annotations correspond to those in the Kyoto Encyclopedia of Genes and Genomes (KEGG). **(B)** High expression of the ribosome pathway shows significant association with lower YC-1 sensitivity. Click here for file

Additional file 6: Table S3 Pathways potentially associated (false discovery rate <5%) with the breast cancer response to YC-1. Click here for file

Additional file 7: Figure S4 Analysis of ERα localization and levels following exposure to YC-1. **(A)** ERα is mislocalized upon exposure to YC-1 in both MCF7 and MCF7-LTED cells. **(B)** Total ERα levels are reduced upon exposure to YC-1 in both MCF7 and MCF7-LTED cells, although relatively more in MCF7-LTED cells. **(C)** Subcellular fractionation does not reveal differences for ERα. Ponceau protein staining and detection of the 62 kDa nucleoporin (NUP62) were used as loading controls. Click here for file

Additional file 8: Figure S5 Expression analysis with exposure to YC-1. **(A)** High expression of the Ribosome pathway (false discover rate <5%) is shown in the parental MCF7. **(B)** Top panels, the Ribosome pathway is significantly altered (that is, underexpressed) in MCF7 cells, but not in MCF7-LTED cells, exposed to YC-1. Bottom panels, both MCF7 and MCF7-LTED cells show underexpression of the cell cycle pathway with exposure to YC-1. **(C)** Western blot analysis results of phospho-serine 235/236 S6 ribosomal protein, E2F1 and control TUBA in MCF7 and MCF7-LTED cells in basal or YC-1-exposed conditions. Click here for file

Additional file 9: Table S4 Pathways differentially expressed (false discovery rate <5%) in MCF7 and/or MCF7-LTED cells, in basal and/or YC-1 conditions. Click here for file

Additional file 10: Table S5 Differential expression analysis of predicted E2F1 target sets (false discovery rate <1%) in MCF7 and MCF7-LTED cells exposed to YC-1. Click here for file

Additional file 11: Figure S6 Results from RAC1 activity assays with depletion and/or reconstitution of MYC-Vav3. Left panel, graph depicting RAC1 activity from triplicate assays in the conditions depicted across the *x*-axis. The asterisks correspond to significant differences (*P* < 0.05). Right panels, Western blot analysis results of total VAV3, MYC (for MYC-Vav3) and control TUBA in MCF7 and MCF7-LTED cells transduced with shRNA control (pLKO.1) or shRNA-*VAV3* plus MYC-Vav3 constructs. Click here for file

Additional file 12: Table S6 Results of the GWAS and the replication study for SNPs in *VAV3*. Click here for file
