# Supplementary material for: VAV3 mediates resistance to breast cancer endocrine therapy
Source: Breast Cancer Res. 2014 May 28;16(3):R53. doi: 10.1186/bcr3664 (PMC4076632; doi:10.1186/bcr3664)
Supplement: Additional file 1: Table S1 — Results from the chemical compound screen. [file bcr3664-S1.pdf]

Table S1 Results from the chemical compound screen

Compounds that showed higher inhibition of MCF7-LTED cells

1. 1-(4-Chlorobenzyl)-5-methoxy-2-methylindole-3-acetic acid
2. 3-Amino-1-propanesulfonic acid sodium salt
3. Amoxapine
4. BMY 7378 dihydrochloride
5. Bromoacetyl alprenolol menthane
6. Cantharidic acid
7. Carisoprodol
8. CL 316.243
9. Cyclosporin A
10. Finasteride
11. γ-Acetylinic GABA
12. Paroxetine hydrochloride hemihydrate
13. YC-1

Compounds that showed higher inhibition of MCF7 cells

1. Retinoic acid, 13-cis-retinoic acid
2. b-Chloro-L-alanine hydrochloride
3. L-765.314 (α1B- Adrenoreceptor inhibitor)
4. Agmatine sulfate
5. R-(-)-Apomorphine hydrochloride hemihydrate
6. Acetamide
