# Supplementary material for: VAV3 mediates resistance to breast cancer endocrine therapy
Source: Breast Cancer Res. 2014 May 28;16(3):R53. doi: 10.1186/bcr3664 (PMC4076632; doi:10.1186/bcr3664)
Supplement: Additional file 2: Table S2 — Values of YC-1 IC50 (μM) in breast cancer cell lines. [file bcr3664-S2.pdf]

**Table S2** Values of YC-1 $IC_{50}$ (μM) in breast cancer cell lines.

| Cell line | ERα | PR | HER2 | $IC_{50}$ |
|---|---|---|---|---|
| AU565 | - | - | + | 1.3 * |
| BT474 | + | + | + | 319 |
| HCC1937 | - | - | - | 253 |
| MCF7 | + | + | -/+ | 131 |
| MCF7-HER2 | + | + | + | 187 |
| MCF7-LCC9 | + | ++ | - | 2.6 * |
| MCF7-LTED | ++ | + | -/+ | 4.9 * |
| MCF7-LY2 | + | ++ | - | 2.3 * |
| MCF10A | - | - | - | 99 |
| MDA-MB-231 | - | - | - | 4,482 |
| MDA-MB-453 | - | - | - | 250 |
| MDA-MB-468 | - | - | - | 49 |
| SKBR3 | - | - | + | 1.6 * |
| T47D | + | + | -/+ | 8.2 * |
| ZR751 | + | - | -/+ | 0.3 * |

* $IC_{50} < 10$ μM

!
