# Supplementary material for: VAV3 mediates resistance to breast cancer endocrine therapy
Source: Breast Cancer Res. 2014 May 28;16(3):R53. doi: 10.1186/bcr3664 (PMC4076632; doi:10.1186/bcr3664)
Supplement: Additional file 12: Table S6 — Results of the GWAS and the replication study for SNPs in VAV3. [file bcr3664-S12.pdf]

Table S6 Results of the GWAS and replication study for SNPs in *VAV3*

		Allele 1/2		Genotype count (event)			Genotype count (no event)			Minor allele frequency (event)	Minor allele frequency (no event)	Hardy-Weinberg equilibrium *P*	Log-rank *P*
				11	12	22	11	12	22				
rs2504469	GWAS	T	C	10	9	11	15	91	104	0.48	0.29	0.64	1.1E-03
	Replication			0	10	6	6	37	46	0.31	0.28	0.27	6.7E-01
	Combined			10	19	17	21	128	150	0.42	0.28	0.87	5.7E-03
rs12142335	GWAS	A	G	0	3	27	0	6	204	0.05	0.01	0.77	3.5E-03
	Replication			0	1	15	0	1	88	0.03	0.01	0.92	1.7E-01
	Combined			0	4	42	0	7	292	0.04	0.01	0.76	4.4E-03
rs4462178	GWAS	T	C	0	3	27	0	6	204	0.05	0.01	0.77	3.5E-03
	Replication			0	1	15	0	1	88	0.03	0.01	0.92	1.7E-01
	Combined			0	4	42	0	7	292	0.04	0.01	0.76	4.4E-03
rs10494076	GWAS	A	G	27	3	0	204	6	0	0.05	0.01	0.77	3.5E-03
	Replication			15	1	0	88	1	0	0.03	0.01	0.92	1.7E-01
	Combined			42	4	0	292	7	0	0.04	0.01	0.76	4.4E-03
rs4466688	GWAS	T	G	10	10	10	18	102	90	0.50	0.33	0.69	4.3E-03
	Replication			2	9	5	6	41	42	0.41	0.30	0.28	2.4E-01
	Combined			12	19	15	24	143	132	0.47	0.32	0.38	4.5E-03
rs2494047	GWAS	T	C	11	9	10	98	93	19	0.48	0.31	0.50	4.4E-03
	Replication			6	7	3	44	39	6	0.41	0.29	0.73	2.0E-01
	Combined			17	16	13	142	132	25	0.46	0.30	0.69	3.2E-03
rs7543435	GWAS	A	G	10	10	10	90	100	20	0.50	0.33	0.98	6.0E-03
	Replication			5	8	3	41	42	6	0.44	0.30	0.37	1.5E-01
	Combined			15	18	13	131	142	26	0.48	0.32	0.63	3.4E-03
rs10494072	GWAS	T	C	17	7	6	137	69	4	0.32	0.18	0.87	7.3E-03
	Replication			9	7	0	63	25	1	0.22	0.15	0.21	3.4E-01
	Combined			26	14	6	200	94	5	0.28	0.17	0.66	9.0E-03
rs4915063	GWAS	A	G	9	11	10	20	97	93	0.48	0.33	0.93	8.2E-03
	Replication			3	8	5	6	42	41	0.44	0.30	0.37	1.5E-01
	Combined			12	19	15	26	139	134	0.47	0.32	0.69	4.3E-03
rs10494071	GWAS	T	C	3	11	16	39	110	61	0.28	0.45	0.64	8.7E-03
	Replication			1	5	10	17	42	30	0.22	0.43	0.51	4.0E-02
	Combined			4	16	26	56	152	91	0.26	0.44	0.98	8.4E-04
rs4914950	GWAS	T	C	3	12	15	42	108	60	0.30	0.46	0.81	1.2E-02
	Replication			1	6	9	18	43	28	0.25	0.44	0.69	5.0E-02
	Combined			4	18	24	60	151	88	0.28	0.45	0.99	1.5E-03
rs6693140	GWAS	T	C	15	11	4	137	66	7	0.32	0.19	0.76	2.5E-02
	Replication			9	7	0	55	33	1	0.22	0.20	0.05	9.0E-01
	Combined			24	18	4	192	99	8	0.28	0.19	0.43	6.4E-02
rs1410406	GWAS	A	G	3	7	20	22	102	86	0.22	0.35	0.70	3.0E-02
	Replication			1	5	10	11	39	39	0.22	0.34	0.66	2.3E-01
	Combined			4	12	30	33	141	125	0.22	0.35	0.93	1.6E-02
rs17019602	GWAS	A	G	16	9	5	128	76	6	0.32	0.21	0.73	3.4E-02
	Replication			8	8	0	60	28	1	0.25	0.17	0.11	2.6E-01
	Combined			24	17	5	188	104	7	0.29	0.20	0.29	2.7E-02
rs11185141	GWAS	T	C	17	10	3	71	110	29	0.27	0.40	0.37	3.6E-02
	Replication			9	6	1	37	37	15	0.25	0.38	0.27	3.1E-01
	Combined			26	16	4	108	147	44	0.26	0.39	0.89	2.1E-02
rs9804074	GWAS	A	G	1	7	22	1	35	174	0.15	0.09	0.88	3.9E-02
	Replication			0	4	12	2	13	74	0.13	0.10	0.30	7.4E-01
	Combined			1	11	34	3	48	248	0.14	0.09	0.65	1.1E-01
rs12746044	GWAS	A	G	4	9	17	5	64	141	0.28	0.18	0.88	4.3E-02
	Replication			0	6	10	1	27	61	0.19	0.16	0.18	8.1E-01
	Combined			4	15	27	6	91	202	0.25	0.17	0.59	8.1E-02
rs9803825	GWAS	A	G	5	8	17	5	74	131	0.30	0.20	0.75	4.4E-02
	Replication			0	8	8	1	28	60	0.25	0.17	0.11	2.6E-01
	Combined			5	16	25	6	102	191	0.28	0.19	0.29	3.2E-02
rs12039907	GWAS	A	G	16	10	4	130	76	4	0.30	0.20	0.27	4.5E-02
	Replication			8	8	0	51	36	2	0.25	0.22	0.05	7.4E-01
	Combined			24	18	4	181	112	6	0.28	0.21	0.05	5.9E-02
rs6583035	GWAS	T	C	20	9	1	169	39	2	0.18	0.10	0.98	4.6E-02
	Replication			13	3	0	68	20	1	0.09	0.12	0.65	6.4E-01
	Combined			33	12	1	237	59	3	0.15	0.11	0.78	2.2E-01
